# An Unusual Culprit Behind Right Lower Quadrant Pain: Cecal Adenocarcinoma Initially Suspected as Appendicitis in an Elderly Female Patient

**DOI:** 10.7759/cureus.90004

**Published:** 2025-08-13

**Authors:** Michail Angelos Papaoikonomou, Europi Michailidou, Aggeliki Chlorou, Nikolaos Krokos

**Affiliations:** 1 Department of General Surgery, Agios Pavlos General Hospital, Thessaloniki, GRC

**Keywords:** acute appendicitis mimic, atypical appendicitis, cecal adenocarcinoma, colorectal surgery, right hemicolectomy

## Abstract

This case report addresses an unusual etiology of right lower quadrant (RLQ) abdominal pain in an elderly patient. While acute appendicitis is a common surgical emergency, especially among young people, older patients may exhibit atypical symptoms that conceal a more severe pathology. The main objective of this article is to highlight the diagnostic challenges and therapeutic implications of cecal adenocarcinoma that mimics acute appendicitis.

We describe the clinical presentation, diagnostic workup, intraoperative findings, and postoperative course of a 74-year-old female patient who had symptoms compatible with acute appendicitis. Despite preoperative imaging indicating appendicitis, the surgical investigation revealed a cecal tumor, prompting a right hemicolectomy. The initial investigation revealed localized RLQ pain and leukocytosis, along with elevated C-reactive protein (CRP). Imaging studies indicated retrocecal inflammation and cecal distention. The intraoperative evaluation revealed a grossly normal appendix but a firm cecal mass. A right hemicolectomy was performed. Histopathology revealed a moderately differentiated cecal adenocarcinoma classified as pT3N0Mx. The patient's recovery went smoothly, and she was discharged on postoperative day 7 with an oncology referral.

Cecal carcinoma can manifest as acute appendicitis in elderly patients, necessitating a high level of suspicion and careful intraoperative evaluation. Prompt conversion to definitive oncologic surgery, as proven, enables correct diagnosis and timely treatment. Surgeons should be extra cautious, especially in patients over 40, and perform appropriate postoperative screening in search of underlying malignancies.

## Introduction

Acute appendicitis is most commonly observed in adolescents and young adults, with a lifetime risk estimated at approximately 7% to 8% and a higher prevalence among males [1-2]. It is classically characterized by periumbilical pain that migrates to the right iliac fossa, often accompanied by anorexia, nausea, vomiting, and low-grade fever. However, the incidence, presentation, and underlying causes of appendicitis change significantly with age. Elderly patients often present atypically [2]. While lower abdominal pain is still common, classic features like fever, localized tenderness, and migratory pain may be less pronounced or absent. This is partly due to decreased pain perception, lower basal temperature/diminished thermoregulatory response, and a blunted inflammatory response in older adults [2]. The most common symptoms associated with acute appendicitis, as observed in a review of elderly patients diagnosed with acute appendicitis, were lower abdominal pain (93.9% to 97.6%), anorexia (57.6% to 67.0%), nausea-vomiting (45.5% to 68.3%), migrating pain (30.3% to 45.1%), right iliac fossa pain (60.6%), and fever (21.2% to 26.8%) [2]. In individuals over 50 years of age, appendicitis becomes relatively uncommon; approximately 90.7% of cases occur before age 65, and only 1.6% after age 80, and the etiologic factors tend to differ from those seen in younger populations [3]. While in younger patients the condition is frequently caused by luminal obstruction due to fecalith or lymphoid follicular hyperplasia (accounting for 50% to 80% of cases), older adults more often experience appendicitis secondary to atypical causes such as gallstones, parasitic infections, or, more concerningly, neoplasms [1, 3]. 

 While typical in younger populations, elderly patients with similar presentations often harbor alternative or more sinister pathology. In elderly patients presenting with right lower quadrant (RLQ) pain, malignancy should be considered in the differential diagnosis, especially when intraoperative findings are atypical or when the appendix appears normal [1]. In such cases, an underlying cecal mass may either mimic appendicitis or coexist with it. Primary tumors of the cecum, particularly adenocarcinomas, can present with signs and symptoms indistinguishable from appendicitis, posing a diagnostic challenge. More rarely, large adenocarcinomas of the colon can present with clinical features mimicking acute appendicitis. This occurs when the mass effect of the tumor leads to luminal obstruction at or near the appendiceal orifice, triggering symptoms similar to those of acute appendicitis [1]. While this presentation is uncommon, it is clinically significant, especially in older adults. Notably, only 8% to 10% of colorectal cancers arise in the cecum, and among these, only a small fraction progress to a stage where they produce appendicitis-like symptoms [1]. Such cases may be misdiagnosed as simple appendicitis unless careful intraoperative or radiologic evaluation is performed [1]. This underscores the importance of maintaining a high index of suspicion and proceeding with further exploration when unexpected findings are encountered during surgery. Early identification is critical for timely surgical intervention and oncologic management. 

Incidental masses or lesions are found in appendectomy specimens with varying incidence depending on the extent of pathological sampling. Studies show that multiple and dense sampling increases detection of incidental lesions, including benign and malignant tumors [4]. Malignant tumors, including neuroendocrine tumors, low-grade mucinous neoplasms, and adenocarcinomas, are found incidentally in about 1% to 2% of appendectomy specimens on average across studies [5-6]. Neuroendocrine tumors, the most common appendiceal malignancy, are incidentally found in approximately 0.3% to 2.3% of appendectomy specimens and can be missed without resection and pathological analysis [4, 7]. Missed malignancy after appendectomy alone is a concern, particularly with non-operative treatment strategies. Appendiceal malignancy may be missed if histopathological examination is not performed or if surgery is not done after conservative treatment [4]. Overall, incidental appendiceal malignancy rates range between roughly 0.8% and 2%, with negative appendectomy (no appendicitis) rates around 9% to 10% in some studies [4, 8]. The risk of delayed or missed diagnosis is higher in older patients and those with complicated appendicitis or appendiceal masses [4].

## Case presentation

A 74-year-old female presented to the emergency department with acute RLQ abdominal pain and fever up to 38°C of 48 hours duration. The pain was associated with nausea but no vomiting. On examination, the patient was febrile, with localized pain, rigidity, and rebound tenderness located over McBurney’s point. Bowel sounds were present in all quadrants. Her past medical history was insignificant other than hypertension and hyperlipidemia. She did not refer to any bowel habit changes nor sudden weight loss for the past months. Her vital signs were normal upon presentation. Laboratory investigations revealed normal hemoglobin (12.6 g/dL) and hematocrit (38.4%), leukocytosis with elevated white blood cell count (13,200/μL), marked neutrophilia (polymorphonuclear predominance 82.3%), and a raised C-reactive protein (CRP) level of 8.8 mg/L. The rest of the laboratory findings are exhibited in Table 1.

**Table 1:** Laboratory investigation findings of our patient upon presentation in the emergency department. It is worth noting that upon the patient's arrival, the laboratory findings indicated an inflammatory condition, and tumor markers were negative.

Parameter	Patient value	Reference range
Leucocytes	13.200/μL	4.200-11.000/μL
Neutrophils (NE%)	82.3%	40-70%
Hemoglobin	12.6 g/dL	14-17.4 g/dL
Hematocrit	38.4%	36- 48%
Platelets	227.000/μL	140.000–390.000/μL
Total bilirubin	0.99 mg/dL	0.3-1.2 mg/dL
Gamma-glutamyl transferase	17 U/L	< 55 U/L (age-dependent)
Aspartate aminotransferase (serum glutamic-oxaloacetic transaminase (SGOT))	15 U/L	5–40 U/L
Alanine aminotransferase (serum glutamic pyruvic transaminase (SGPT))	9 U/L	7–56 U/L
Alkaline phosphatase	69 U/L	44–147 U/L
Creatinine	0.57 mg/dL	0.6-1.3 mg/dL
Carcinoembryonic antigen (CEA)	3.94 ng/mL	< 5.0 ng/mL
Cancer antigen (CA) 19-9	<2.06 U/mL	< 37 U/mL
Alpha-fetoprotein	2.26 ng/mL	< 10 ng/mL
C-reactive protein (CRP)	8.8 mg/L	< 0.5 mg/L

**Figure 1:**
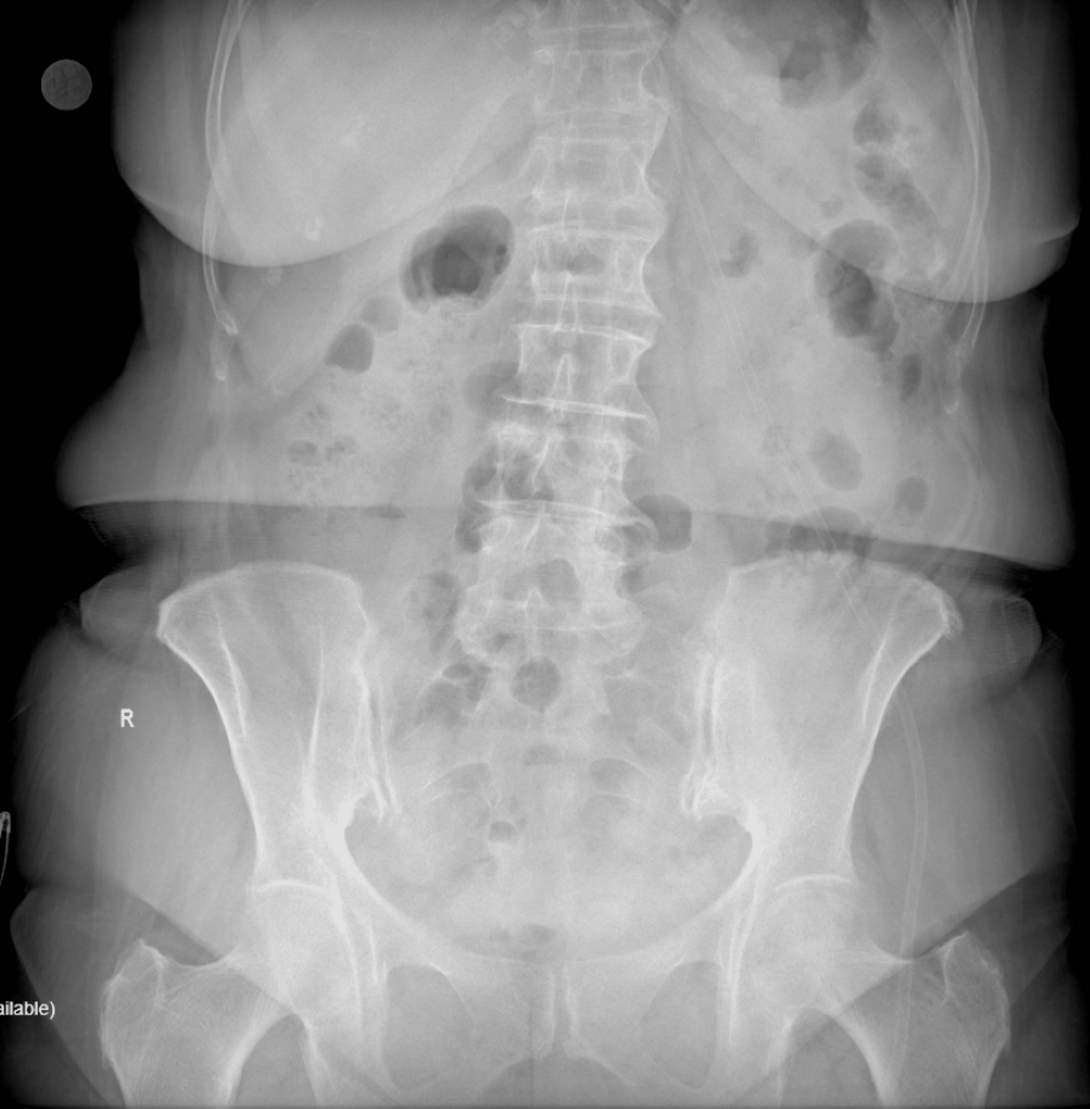
An abdominal X-ray upon presentation in the emergency department showed no dilated bowel loops nor air-fluid levels present.

No abnormal findings were noted on the abdominal X-ray (Figure 1).

**Figure 2:**
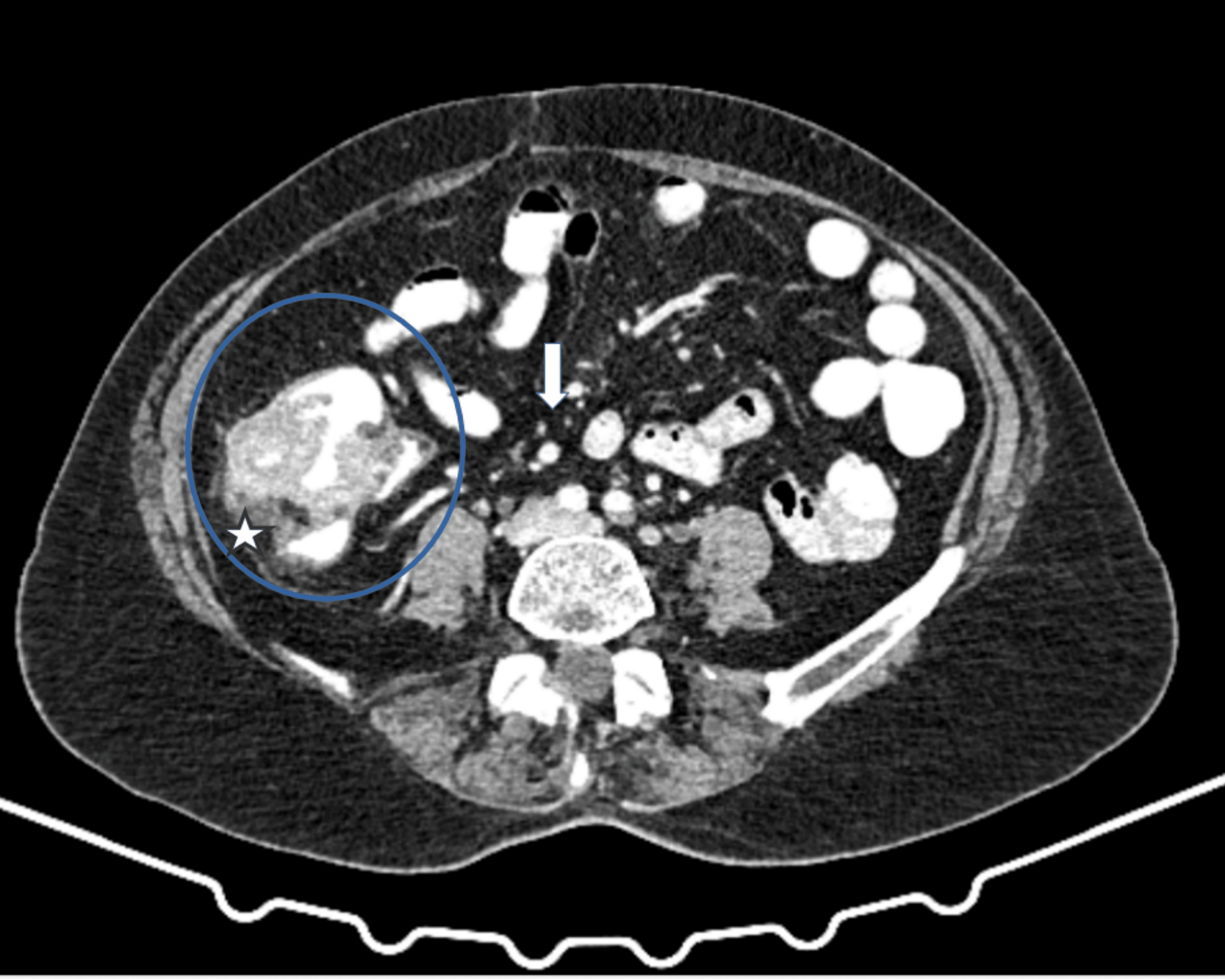
A CT scan of the abdomen with intravenous contrast (axial view) demonstrated cecal dilatation with associated fecal impaction (blue circle). The pericolic fat, particularly in the post-cecal region, showed stranding suggestive of localized inflammation (white asterisk). Additionally, a few enlarged lymph nodes were visible, raising suspicion for reactive lymphadenopathy (white arrow).

The patient was subjected to a CT scan of the abdomen, which showed fluid micro collections with fat stranding located retrocecal, suggesting acute appendicitis. In addition, the cecum appeared distended with many intraluminal filling defects that can be attributed to fecal impaction (Figure 2). 

Given the clinical suspicion of acute appendicitis based on the laboratory findings and the clinical presentation, the patient was admitted to the surgical department and taken to the operating room for an emergency appendectomy. A McBurney incision was made, and the appendix was inspected; it appeared grossly normal. However, intraoperative palpation of the cecum revealed a tumor-like firm growth with a distended terminal ileum, raising suspicion for an underlying neoplastic process. In light of these unexpected findings, the procedure was converted to an exploratory laparotomy. Upon further inspection of the RLQ, the presence of a cecal mass was confirmed that extended to the serosa and lateral abdominal wall. A right hemicolectomy was performed with primary ileocolic anastomosis. The patient tolerated the procedure well and was transferred to the recovery room in stable condition.

**Figure 3:**
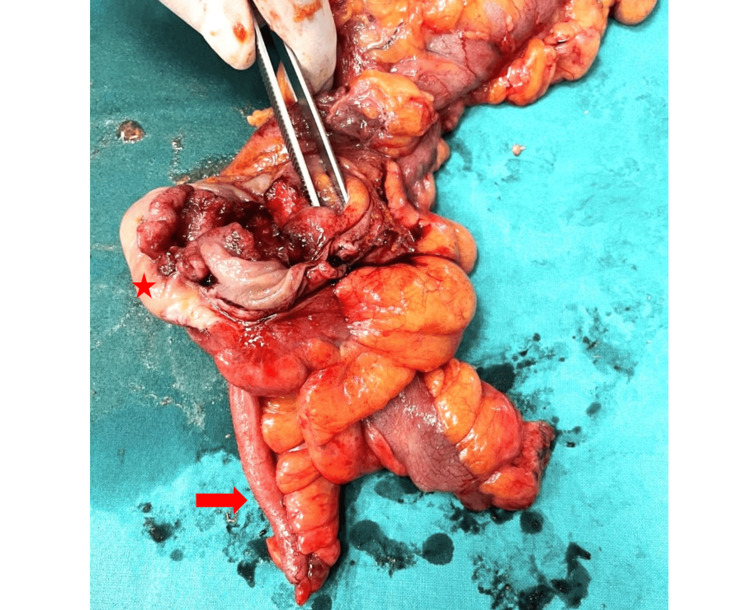
The surgical specimen obtained from the right hemicolectomy Note the circumferential pattern of infiltration of the tumor (red asterisk) and the presence of necrosis/erosion of the colonic wall. The appendix appeared normal during macroscopic evaluation (red arrow).

Gross examination of the surgical specimen revealed an ulcerative lesion in the cecum measuring approximately 4.5 cm in diameter with necrotic foci. The tumor was found to infiltrate the entire thickness of the colonic wall and extend into the pericolic adipose tissue (Figure 3). 

**Figure 4:**
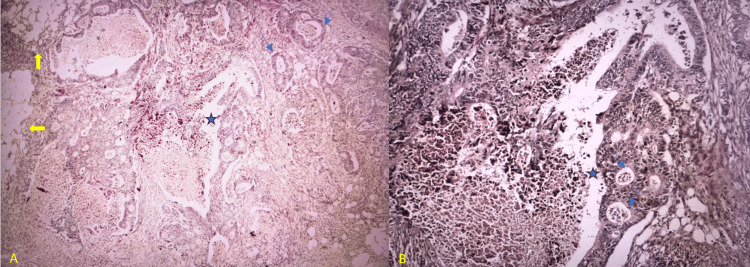
Histopathological analysis of the surgical specimen with hematoxylin and eosin stain (HE). Both slides depicted the adenocarcinoma of the cecum with prominent well-formed glands (blue arrows) filled with necrotic debris (blue asterisk). The tumor was staged as T3 due to the presence of infiltration in the pericolic adipose tissue (yellow arrows). A. x4 magnification; B. x10 magnification

Microscopic analysis confirmed the diagnosis of moderately differentiated adenocarcinoma of the cecum. Tumor invasion was noted through the muscularis propria into the pericolic fat, which was classified, according to the American Joint Committee on Cancer (AJCC) Eighth Edition, as pT3 (Figure 4) [9]. 

Lymphovascular invasion was not present. Regional lymph node dissection yielded 11 nodes, of which none were positive for metastatic disease (pN0). Surgical resection margins were free of tumor involvement. Thus, the tumor was classified as pT3N0Mx. In addition, a polyp measuring 0.6 cm in diameter was identified 4.5 cm distal to the primary tumor. Histological analysis confirmed the polyp as a tubular adenoma with low-grade epithelial dysplasia and no evidence of malignant transformation. The appendix appeared grossly normal intraoperatively. Histological examination showed no evidence of acute or chronic inflammation. However, hyperplasia of tubular glands was present, consistent with reactive changes. 

The patient's postoperative recovery was uneventful. She was managed with intravenous fluids, proton-pump inhibitors, analgesia, and prophylactic antibiotics (piperacillin/tazobactam 4.5 g 1x3 for seven days and amikacin 500 mg 1x2 for five days). Bowel sounds gradually returned, and she passed flatus by the third postoperative day. On postoperative day 4, a fluid diet was initiated, which she tolerated well. Her diet was progressively advanced without complications. Wound healing was satisfactory, and there were no signs of infection or anastomotic leak. She was discharged in stable condition on postoperative day 7 with instructions for outpatient follow-up and referral to oncology for further management.

## Discussion

Colorectal carcinoma is the second leading cause of cancer-related deaths worldwide. Melena, anemia, fatigue, and abdominal discomfort are common symptoms of carcinomas located in the right colon and the cecum. In other cases, the clinical signs include a growth in the right iliac fossa, which may be the apex of an intussusception causing intermittent obstruction that can be discovered during an appendectomy [10]. According to the literature, the occlusion of the appendix by a tumor manifests as a result of three distinct pathophysiological pathways: 1. a colonic tumor that is creating back pressure on the cecum leading to the obstruction of the appendix, 2. a tumor that is in close proximity to the appendiceal orifice, or 3. inflammatory alterations and edema of a cecal carcinoma that causes the occlusion of the appendix [11]. In this case, abdominal pain and rigidity detected during examination served as alarming indicators of an inflammatory reaction. The mass's localization and infiltration of the abdominal wall resulted in rigidity and rebound discomfort, warranting additional investigation. 

Several studies and case reports indicate that appendicitis secondary to cecal adenocarcinoma is rare, accounting for less than 2% of appendicitis cases overall. For example, one review of 218 elderly patients found that only 1.8% of appendicitis cases were due to cecal carcinoma [12-13]. Another study noted that colorectal cancer was diagnosed in about 0.85% of appendicitis patients, with a significant increase to around 1.76% in those over 40 years old [13]. Thus, the associated diagnostic delay can lead to major implications for patient outcomes. Right-sided colonic malignancies can mimic acute appendicitis, particularly in elderly patients. Appendicitis presenting in any individual older than the age of 40 should raise clinical suspicions for the possibility of malignancy [14]. Therefore, any patient older than 40 who underwent a traditional appendectomy should be highly recommended to schedule a follow-up colonoscopy six weeks postoperatively. A systematic review including nearly 4,000 patients found that post-appendectomy colorectal screening in patients over 40 years detected lesions in 24% and colorectal cancer in about 4%, with nearly half of the cancers located in the right colon or cecum, areas closely related to appendicitis presentations [15]. Screening colonoscopy in patients aged 40 and above after appendicitis showed a 15% adenoma detection rate, supporting the notion that appendicitis in this age group may be a marker for underlying colorectal neoplasia [16]. 

Imaging, while useful, may not reliably distinguish between inflammatory and neoplastic etiologies [14]. While CT scans may reveal appendicitis, the underlying cecal mass may not always be apparent, particularly in the setting of acute inflammation [10, 14]. Imaging findings (especially CT) that suggest acute appendicitis can also be caused by right-sided colon cancer, as peri-appendiceal fluid and appendiceal dilatation may result from partial or low-grade obstruction by a colonic mass [14]. In other instances, a mass is only detected during surgery or on postoperative pathology, as in our case. Intraoperative findings should prompt appropriate oncologic resection when malignancy is suspected. This case underscores the importance of maintaining a high index of suspicion for colorectal cancer in older patients presenting with appendicitis-like symptoms. In our case, an immediate right hemicolectomy was conducted, taking into account the extent of colonic involvement and the uncertain nature of the cecal mass. Although some studies advocate for preoperative biopsy for confirmation of malignancy or advanced imaging before undertaking definitive oncologic resection, such an approach is not always feasible in emergent settings. In elderly patients, delays in surgical management, particularly in the context of a suspicious mass with inflammatory and/or obstructive characteristics, carry a significant risk of perforation, peritonitis, and increased postoperative morbidity [17]. Therefore, proceeding directly to right hemicolectomy was considered both a diagnostic and therapeutic intervention, consistent with established surgical principles in cases where malignancy cannot be excluded intraoperatively.

This case underscores the critical importance of maintaining a high index of suspicion for underlying malignancy in older adults presenting with signs and symptoms of acute appendicitis. While appendicitis is typically benign in younger patients, in elderly individuals, particularly those with risk factors such as obesity, smoking, sedentary lifestyle, or known genetic and acquired mutations, the differential diagnosis must be broadened to include malignancies [1]. In our case, the patient had no familial or personal history of malignancy and followed a relatively good sedentary lifestyle. Clinical scoring systems commonly used in younger patients (e.g., Alvarado score) are less reliable in the elderly, and diagnosis should rely on a combination of clinical suspicion, laboratory tests, and imaging [2]. Even when the clinical picture appears straightforward, surgeons and clinicians must remain vigilant for atypical findings and must be prepared to escalate the intervention appropriately. The differences associated with the clinical presentation, etiology, and general trends of acute appendicitis in old and young patients are exhibited in Table 2 [1-3]. 

**Table 2:** A summary of the clinical features and outcomes in younger and older patients with acute appendicitis Source: [1-3]

Feature	Younger patients	Older patients (>50 years)
Incidence	High	Low
Typical cause	Lymphoid hyperplasia, fecalith	Fecalith, fibrosis, neoplasia, gallstones
Classic presentation	Migratory pain, fever, anorexia	Atypical, less fever, vague pain
Risk of complications	Lower	Higher (perforation, abscess)
Malignancy risk	Low	Higher; malignancy must be excluded
Diagnostic challenges	Less	More (atypical, delayed diagnosis)

## Conclusions

This case highlights the diagnostic challenge posed by a cecal mass presenting as acute appendicitis in an elderly patient. As a consequence, patients with missed or delayed cancer diagnoses experience poorer outcomes and are prone to life-threatening complications. Therefore, when dealing with acute appendicitis in older adults, especially in advanced age, the possibility of a malignant tumor should always be considered in the differential diagnosis. Right hemicolectomy enabled both definitive diagnosis and appropriate oncologic management in this patient. Prompt recognition, thorough intraoperative evaluation, and appropriate postoperative follow-up play a key role in improving prognosis and reducing the burden of undiagnosed colorectal cancer in patients presenting with appendicitis, particularly those over 40 years of age.
